# Impact of soil and water conservation practices on crop income in tembaro district, southern Ethiopia

**DOI:** 10.1016/j.heliyon.2022.e10126

**Published:** 2022-08-10

**Authors:** Seyfu Tesfayohannes, Getahun Kassa, Yared Mulat

**Affiliations:** a Areka Agricultural Research Center, Southern Agricultural Research Institute, P.O. Box 79, Areka, Ethiopia; b Wondo Genet College of Forestry and Natural Resources, Hawassa University, P.O. Box 128, Shashemene, Ethiopia

**Keywords:** Average treatment effect, Crop income, Propensity score matching, Soil conservation practice

## Abstract

This study investigated the impact of soil and water conservation practices on crop income in the Tembaro district, Kembata Tembaro zone, Southern Ethiopia. We selected 236 households using stratified sampling. For this study, we collected primary data through structured questionnaires, focus group discussions, and interviews with key informants. Propensity score matching was used to investigate the impacts of soil conservation initiatives on agricultural income. Age, distance from the farmer’s training center, total land size, extension contact, and training all influence participation in soil and water conservation practices. ATE revealed that crop income differed positively between the control and treatment groups. The total household income increased by 422 ETB as a result of participation in the program. This demonstrates the importance of soil and water conservation for boosting crop income. As a result, governmental and non-governmental development partners should invest in farmer capacity building through extension and training to achieve soil and water conservation goals while simultaneously addressing the livelihood issues of resource-dependent local farmers.

## 1 Introduction

### 1.1 Background of the study

Soil erosion and degradation limit food production, pose a threat to climate change and human health and pollute the air and water quality by releasing particles, sediments, and nutrients (FAO, 2019). Every year, soil erosion destroys approximately 10 million acres of agricultural land, decreasing the quantity of cropland available for food production (Pimentel, 2006). Soil deterioration is estimated to cost 0.41 percent of the global GDP each year (Nkonya et al., 2016). Environmentally responsible agricultural practices such as soil and water conservation appear to be a step in the right direction to meet global food demands in a more environmentally sustainable manner (Fontes, 2020). Without soil and water conservation regulations, the cost of remediating soil degradation would increase, productivity would continue to decline, lower agricultural export revenue, and increase food insecurity (Darkwah et al., 2019).

Soil degradation is one of the most serious environmental problems in Ethiopia. Every year, Ethiopia loses almost two billion tons of soil, with cultivated land accounting for half of it (FAO, 2019). Soil and water conservation is a technique for reducing soil and water degradation and increasing crop yields (Sileshi et al., 2019). To stop the decline and increase productivity, integrated soil and water conservation is often implemented (Erkossa et al., 2018). Soil and water conservation methods are viewed as solutions to strengthen the resilience of agriculture to climate change (Fontes, 2020).

In Ethiopia, where soil deterioration has been a long-standing challenge affecting sustainable land use and national food security, research has focused on the efficacy of soil and water conservation techniques (Fontes, 2020). Studies have indicated that watershed development intervention activities lead to improved agricultural productivity, job opportunities, household income, and food security (Ayalew, 2011; Kassa et al., 2013; Tang et al., 2013; Datta, 2015; Nyssen et al., 2015; Yaebiyo et al., 2015; Gebregziabher et al., 2016; and Meaza et al., 2016; Siraw et al., 2020). However, detailed empirical research on the economics of soil conservation is scarce (Kassie et al., 2008). Although the Southern Agricultural Research Institution (SARI) initiated a watershed-based food security effort in Tembaro in 2017, the impact of these measures on the area, which is expected to increase farm production and crop income, is yet to be assessed. The goal of this research is to determine how soil and water conservation techniques affect agricultural profitability in the Tembaro district of Southern Ethiopia’s Kembata Tembaro zone.

## 2 Research methodology

### 2.1 Description of the study area

**Figure 1:**
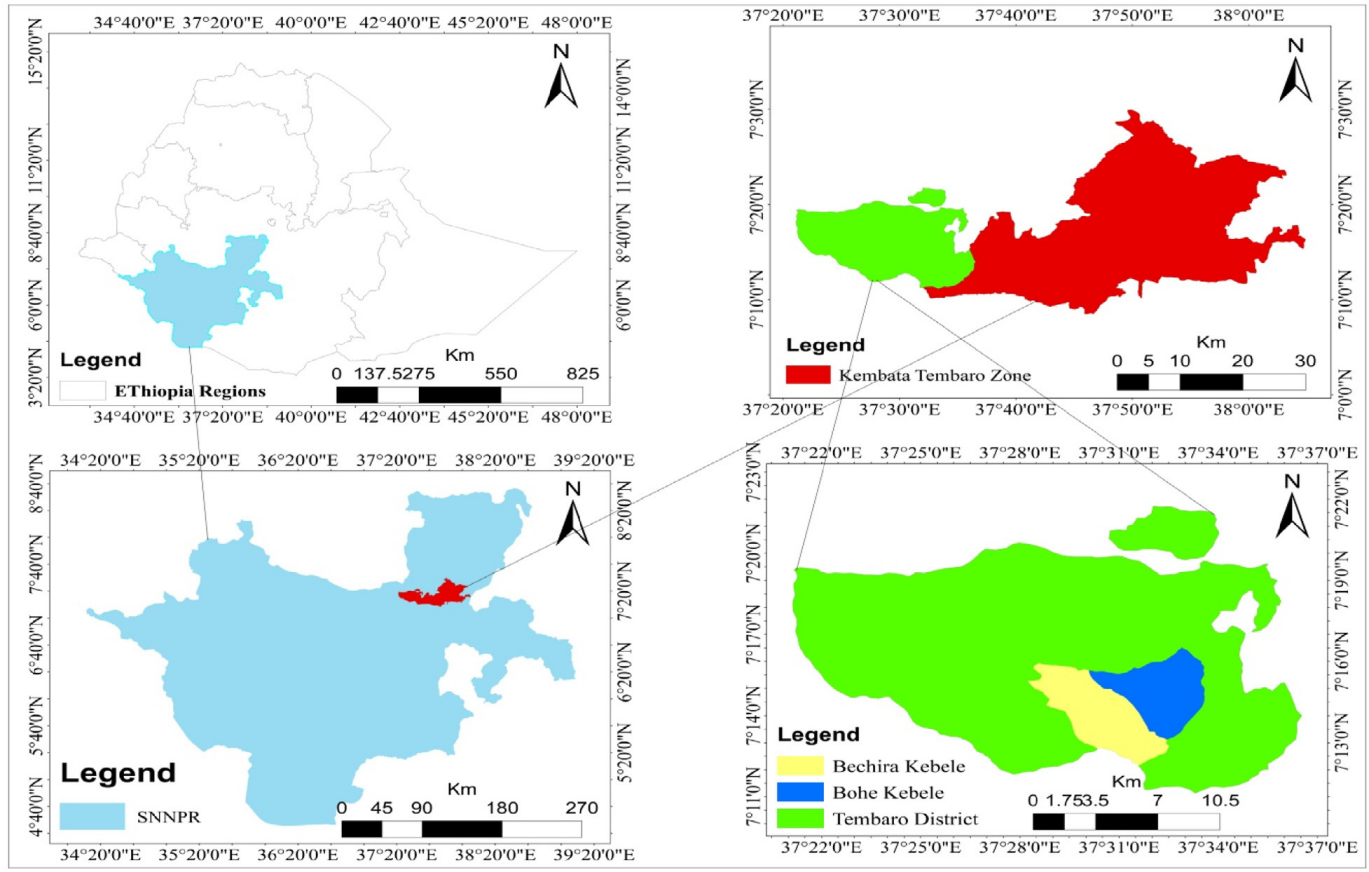
Map of the study area.

This research was conducted in the Tembaro area of southern Ethiopia’s Kembata Tembaro zone. It is 410 km south of Addis Ababa (Figure 1). The district’s overall size is estimated to be 27,917 square kilometers, with two urban and twenty-one rural kebeles. The altitude of the district ranges from 1420 to 2800 m a. s. l.

### 2.2 Sampling procedure and sample size determination technique

In this study, we used a multi-stage sampling method. The study district was selected because the Southern Agricultural Research Institute is currently working on a project in this area. Two kebeles from the district were chosen based on project implementation. Subsequently, households in the chosen kebeles were split into two groups. The stratum represents the treatment group, whereas the stratum two represents the control group. Using a simple random selection technique, 236 representative sample households (111 program participants and 125 non-participants) were selected for the final phase (Table 1).

**Table 1:** Sample size determined for the study.

Sample kebeles	Total households	participant	Non-participant	Sample participant	non-participant	Total sample
Bohe	1138	605	533	71	62	133
Bechira	884	342	542	40	63	103
Total	2022	947	1075	111	125	236

A simplified formula from Kothari (2004) was used to determine the sample size**.**

$$\text{ ​n}=\frac{{\text{z}}^{2}\text{pqN}}{{\text{e}}^{2}\left(\text{N}-1\right)+{\text{z}}^{2}\text{pq}}$$

Where n = the smallest number of sample sizes within the allowed error margin range.
N = the total number of target population households (2,022).
z = confidence level (95 percent, which is equivalent to 1.96).
e = acceptable error margin (6%);

p and q indicate the proportion of program participants and non-participants, respectively, with a 50% probability.

As households were not evenly distributed among the kebeles, the sample size for each kebele was calculated as follows:

$$\;\text{ ​ni}=\frac{\text{Ni}}{\text{N}}\left(\text{n}\right)$$

Where ni = the required sample size from each kebele.
Ni = total number of households in each kebele (participants and non-participants).
N = total number of households in all kebeles.

### 2.3 Methods of data collection

Primary data were gathered through a formal survey, key informant interviews, and focus group discussions. In the two focus groups, model farmers, youths, and female households were selected from each kebele. District and kebele officials, as well as development agents with comprehensive knowledge of soil conservation techniques and contemporary conservation trends, were included in key informant interviews. The household survey was conducted by qualified enumerators from the district agricultural office who was familiar with the local culture and language. This study received ethical approval from the Wondo Genet College of Forestry and Natural Resources of Hawassa University. Informed consent was obtained from all individuals for interviews with human participants.

### 2.4 Methods of data analysis

#### 2.4.1 Descriptive statistics

The mean, Pearson’s chi-squared (χ2) test, and t-test were employed to check for statistical differences in the socio-demographic variables between the treatment and control groups.

#### 2.4.2 Model specification for propensity score matching

Propensity score matching was used to adjust for potential baseline confounders between groups based on sex, marital status, age, family size, education, experience, distance from farmers’ training centers, livestock holding, land holding, social position, frequency of extension contact, use of credit, and training. A logistic regression model was used to determine the propensity scores of the two groups (treatment and control groups).

The following relationships characterize the logit model:

$$Li=\ln\left(\frac{pi}{1-pi}\right)=Zi=\beta 1+\beta 2Xi+Ui$$

Where pi is the likelihood of taking part in the SWC practice.

1-Pi is the likelihood that a household does not participate in the program.

Xi represents the number of explanatory variables that influence involvement in SWC practice.

The estimated propensity score e (xi) for subject i (i = 1... N) is the conditional likelihood of being assigned to a specific treatment, given a vector of observable covariates xi.

e(xi)=Pr(zi=1|xi)

and

$$\mathrm{Pr}\left(z1,\ldots ,\;z\text{n}|\text{x}1,\ldots ,\text{xn}\right)=\begin{array}{l} \text{n}\\ \text{Õ}\\ \text{i}=1 \end{array}e{\left(xi\right)}^{zi}{\left\{1-e\left(xi\right)\right\}}^{1-zi}$$

where: zi = 1, for the treatment.

zi = 0, for control
xi, the vector of observed covariates for the ith subject

The propensity scores ranged from 0 to 1. All statistical analyses were performed using STATA 14.2.

## 3 Results and discussion

### 3.1 Demographic and socioeconomic characteristics of households

Tables 2 and 3 show the socioeconomic characteristics of the program participants and the non-participating households. The results demonstrate that male-headed households account for 91.5 percent of the sample households, whereas female-headed households account for only 8.5 percent. In terms of marital status, the findings show that 89 percent of the sample homes were married, while 11 percent were single (divorced and widowed). 68.47% of the program participants could read and write, while 31.53 percent couldn’t. In comparison, 64% of the program non-participants could read and write, whereas 36% were unable to do so. In terms of the frequency of extension service visits, 46 percent of the program participants and 21% of the non-participants were regularly visited by extension workers.

**Table 2:** Characteristics of households with program participation.

Variables	Category	Participant		Non-participant		Total Sample		χ 2-value
		No Percent		No Percent		No Percent		
Sex	Male		88.3	118	94.4	216	91.5	2.0409
	Female	13	11.7	7	5.6	20	8.5	
Marital_ status	Married	97	87.4	113	90.4	210	89	0.6312
	Single	14	12.6	12	9.6	26	11	
Education	Read & Write	76	68.47	80	64	156	66	0.5293
	Cannot read	35	31.53	45	36	80	34	
Social position	position	19	17.1	15	12	34	14.4	2.0349
	no position	92	82.9	110	88	202	85.6	
Training	Yes	78	70.3	31	24.8	109	46.2	13.83∗∗∗
	No	33	29.7	94	75.2	127	53.8	
Credit	Yes	17	15.32	18	14.4	35	15	
	No	94	84.68	107	85.6	201	85	0.0005
Extension contact	>20	51	46	26	21	77	33	
	<20	60	54	99	79	159	67	15.26∗∗∗

Note: - ∗∗∗ statistically significant at 1%.

**Table 3:** Characteristics of household and program participation (continuous variables).

Variables	Participant		Non-participants		Total Sample	t-test
	Mean	SD	Mean	SD		
Age	44.55	10.1	43.69	8.69	43.94	−2.15∗∗
Family size	7.32	2.002	6.89	1.89	6.91	1.23
Land size	1.13	0.76	1.01	0.57	1.05	5.59∗∗
TLU	2.069	1.07	1.84	0.999	1.88	3.22 ∗∗∗
Crop Income	15,978.76	7,718.66	10,303.5	5,974.59	12,942.59	3.50∗∗∗
Distance_FTC	2.4	0.924	2.93	1.74	2.65	−2.00∗∗∗
Farm experience	22.72	9.41	23.000	8.02	22.89	−0.46

∗∗∗ and ∗∗ statistically significant at 1% and 5% respectively.

The heads of the sample households were 43.94 years old. Regarding household size, the average number of family members is 6.91. Participants owned an average of 1.13 ha of land, while non-participants owned an average of 1.01 ha. The average total livestock unit (TLU) of the sample households was 1.88. The average annual household income was 12,942.59 ETB.

#### 3.1.1 Crop yield

The main crops grown in the study region during the 2019/20 agricultural season are listed in Table 4. Teff yielded 12.2 quintals per hectare, whereas sorghum yielded 19.94 quintals per hectare. Non-participants produced 10.71 qt/ha teff and 18.4 qt/ha sorghum, while participants harvested 13.27 qt/ha teff and 21.63 qt/ha sorghum. On average, those who participate in soil and water conservation programs produce higher levels of agricultural production than those who do not. This is because the soil and water conservation programs in the treatment group preserved more soil nutrients than those in the control group did. Birtukan et al. (2020) found that watershed management interventions boosted crop yield and revenue, which is consistent with our findings. A recent review by Wolkaa et al. (2018) indicated that the observed yield improvement from SWC is mainly due to the retention of soil moisture, nutrients, and SOC.

**Table 4:** Major crop production in the study area.

Variable	Teff			Sorghum		
	Area (ha)	Production (qt)	Yield (qt/ha)	Area (ha)	Production (qt)	Yield (qt/ha)
Program Participant	0.52	6.9	13.27	0.202	4.37	21.63
Non-Participant	0.42	4.5	10.71	0.17	3.12	18.35
Total	0.48	5.85	11.99	0.19	3.79	19.99

### 3.2 The economic impact of soil and water conservation on crop income

#### 3.2.1 Determinants of participation in soil and water conservation

The results of the logistic regression model investigation of the factors influencing farmers’ participation in soil and water conservation initiatives are presented in Table 5. The expected values fit the observed data reasonably well, according to the results of the binary logistic regression model. The estimated LR^2^ test result is 83.89, suggesting that the coefficient of, at least, one predictor is not equal to zero. Furthermore, the complete model, including all predictors, was highly significant (Prob >2 (DF = 13), p = 0.000).

**Table 5:** Logit model result of household program participant and non-participant.

Independent Variables	Coef.	Std. Err.	Z	P > z	Odds Ratio
Sex	−1.30872	0.976955	−1.34	0.18	0.270165
Age	−0.05077	0.02594	−1.96∗∗	0.050	1.052081
Marital_status	0.641198	0.814803	0.79	0.431	1.898755
Farm size	0.115353	0.093792	1.23	0.219	1.12227
Education	−0.02307	0.042184	−0.55	0.584	0.977193
Farm experience	−0.02415	0.022145	−1.09	0.276	0.976144
Distance_FTC	−1.44488	0.615116	−2.35∗∗	0.019	0.235775
TLU	0.59885	0.20258	2.96∗∗∗	0.003	1.820032
Land size	1.20958	0.263758	4.59∗∗∗	0.000	0.298323
Social position	0.295548	0.352963	0.84	0.402	1.343862
Extension contact	1.658489	0.379699	4.37∗∗∗	0.000	5.251368
Credit	0.098226	0.474377	0.21	0.836	1.103212
Training	0.000338	6.82E-05	4.96∗∗∗	0.000	1.000338
_cons	−2.28681	1.292385	−1.77	0.077	0.101591
Logistic regression	Number of obs = 236				
	LR chi2 (13) = 83.89				
	Prob > chi2 = 0.0000				
Log likelihood = −107.44253	Pseudo R^**2**^ = 0.2808				

∗∗∗ and ∗∗ significant at 1% and 5% respectively. Note: Participation is the dependent variable.

**Age of household head (Age)**: - The age of the household head was linked to soil and water conservation practices negatively and substantially. Despite years of agricultural knowledge, farmers become more risk-averse as they age, making it difficult for them to implement soil and water conservation methods. When all other variables were held equal, a one-year increase in age reduced the involvement in soil and water conservation practices by 1.05 units. This finding is consistent with those of Asfaw and Neka (2017). Similarly, Daniel and Mulugeta (2017) reported that older farmers became exhausted and were unable to take care of their farmlands. In contrast to our findings, Mengistu and Assefa (2020) discovered a favorable association between age and SWC practice.

**Distances from a farmer’s home to farmers’ training center (Distance to FTC):** At a 5% level of significance, the distance between the farmer’s residence and the farmers’ training facility (FTC) negatively influences the involvement of soil and water conservation practices. Thus, living further from FTC decreases the likelihood of engaging in soil and water conservation practices. When the distance between a household’s home and the FTC is increased by one kilometer, the likelihood of participation in soil and water conservation practices decreases by 76.4 percent, whereas other factors remained unchanged. This is because technology transmission and communication at training facilities located far from homesteads requires more time and energy. This finding is consistent with those of Tiwari et al. (2008), Asfaw and Neka (2017), and Wordofa et al. (2020).

**Tropical Livestock Unit (TLU):** This variable is significantly and positively associated with soil and water conservation. Keeping all other variables equal, the odds ratio shows that for every extra increase in cattle, the likelihood of a household participating in soil and water conservation practices improves by 1.82 units. This observation is consistent with the results of other studies (Mengistu and Assefa, 2020; Belachew et al., 2020; Amsalu and De Graaff, 2007). They discovered that having livestock is linked to the adoption of soil and water conservation strategies. According to Belachew et al. (2020), the money earned from the sale of livestock is important for renting labor for soil bund construction. In contrast, Sileshi et al. (2019) discovered that families with higher animal holdings were less likely to employ soil bunds and bench terracing than those with smaller livestock holdings.

**Total land size (land size):** There is a direct link between land size and engagement in soil and water conservation. When other parameters are held constant, the odds ratio reveals that a 1-ha increase in farm size improves the probability of participating in soil and water conservation practices by 70.2 percent. This finding shows that farmers with larger farms were more likely to undertake soil and water conservation than farmers with small plots of land. Therefore, farmers with limited resources are less likely to pursue soil and water conservation. This is because SWC techniques utilize some of the farm’s fertile land, and farmers with larger farms can afford to apply SWC techniques more than those with smaller farms can. Kassa et al. (2013), Woldemariam and Gecho (2017), and Wordofa et al. (2020) have reached similar conclusions. Wordofa et al. (2020) confirmed that farmers with larger farm plots were more likely to be able and willing to use improved SWC measures to reduce land degradation in plots located in sloppy areas. Darkwah et al. (2019) found that farmers in Ghana with larger farms have more financial resources and acreage available to them to promote technology adoption.

**Extension contact (Extension contact):** Participation in soil and water conservation practices is directly related to the frequency of extension. The odds ratio shows a 5.25 difference in the frequency of extension in favor of program participants over non-participants (at a 1% significance level). This means that the farmers’ understanding of physical and biological soil conservation practices has broadened through extended contact. According to Abebe and Bekele (2014), Sileshi et al. (2019), and Wordofa et al. (2020), households with more extension contact and services have a better grasp of the land degradation problem and consider soil and water conservation activities as solutions.

**Training:** Farmers with access to training are eager to adopt soil and water conservation practices. This shows a strong and positive relationship with program participation, implying that households that received training were more likely to undertake soil and water conservation. This is possible because training improves the soil and water conservation knowledge and skills of the farmers. According to various experts, training has a positive and significant impact on participation in soil and water conservation efforts (Tiwari et al., 2008; Asfaw and Neka, 2017; Chesterman et al., 2019; Mengistu and Assefa, 2020). Darkwah et al. (2019) indicated that farmers could be trained to raise awareness and support the adoption of modern technologies. According to Njenga et al. (2021), training provides farmers with a platform for queries and justifications.

#### 3.2.2 Estimation of propensity score

**Figure 2:**
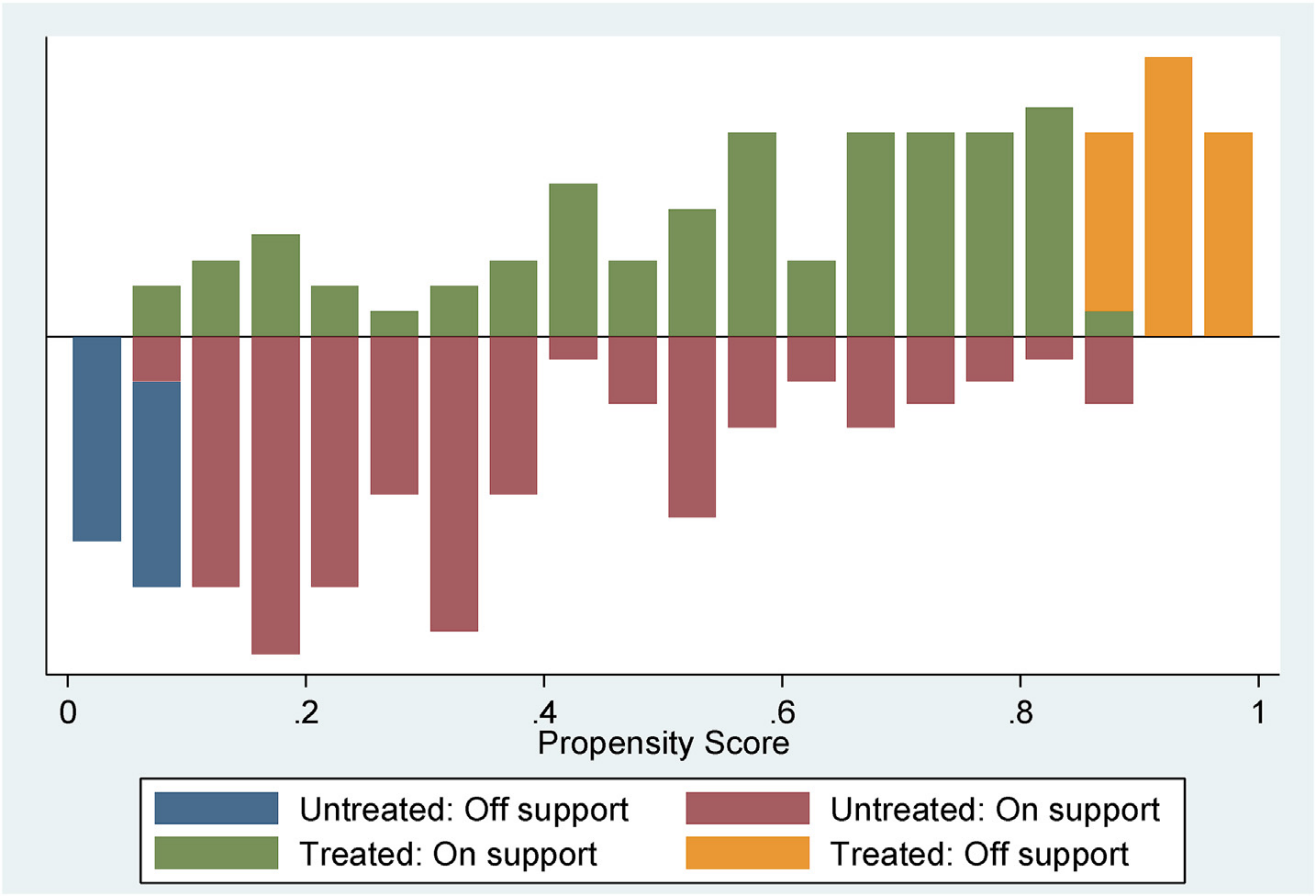
Histogram of the propensity score estimation distribution after matching.

The conditional independence assumption was used to determine the common support regions for the propensity scores of the two groups. The treatment group’s estimated propensity scores ranged from 0.0698221 to 0.9960728, with a mean of 0.6590665, whereas the control group’s scores ranged from 0.0084347 to 0.8538807, with a mean of 0.2963499. Based on the maximum–minimum criteria, the matching technique rejected households with values between 0.0698221 and 0.8538807. Because they were not part of the common support region, 46 observations (30 participants and 16 non-participants) were omitted from the study on the impact of soil and water conservation program participation on crop income. The histogram in (Figure 2) shows the expected common support regions after the matching.

#### 3.2.3 Matching algorithm selection

Propensity score and covariance tests were used to verify whether the matching estimators adequately balanced all explanatory factors for the three matching algorithms (Caliper-matching, Kernel matching, and Nearest Neighbor matching). According to Caliendo and Kopeinig (2008), kernel matching with a bandwidth of 0.25 is the best estimator because it produces a relatively large matched sample size (190), minimum mean bias (4.4), low pseudo-R^2^ (0.012), and best balancing test (all explanatory variables are insignificant after matching) (Table 6). As a result, we estimated the ATE of participation in SWC practices on crop income using a Kernel matching algorithm.

**Table 6:** Comparison of the matching estimators by performance criteria.

Performance criteria					
Algorithm	Estimators (bandwidth)	Matched sample size	Mean Bias	pseudo R^2^	insignificant variables
Caliper-Matching (CM)	0.01	110	16.2	0.155	12
	0.1	144	8.6	0.062	13
	0.25	150	8.1	0.092	13
	0.5	169	10.9	0.148	12
Kernel Matching	0.01	144	10.7	0.052	13
	0.1	190	4.8	0.022	13
	**0.25**	**190**	**4.4**	**0.012**	**13**
	0.5	190	9.3	0.073	13
Nearest Neighbor Matching	1	190	9.9	0.044	12
	2	190	7.9	0.039	13
	3	190	6.4	0.017	13
	5	190	7	0.014	13

#### 3.2.4 Estimating treatment effect on treated (impacts of a program on crop income)

The impact evaluation of the average treatment effect on treated participation in the soil and water conservation program estimated by the kernel matching algorithm was approximately 422 ETB (Table 7). However, after matching, the difference in crop income owing to household program participation was not statistically significant between the two groups. This could be because of the small sample size or the fact that soil and water conservation measures take time to produce results. The changes in household agricultural output and gross crop revenue as a result of soil and water conservation programs are not statistically significant according to Abebe and Bekele (2014), which is consistent with our findings. Similarly, compared to what is typically claimed in favor of watershed development, improvements in terms of economic parameters, such as unit net returns to cultivation, unit sales for farm households in the treated micro-watershed, and improvement of ecologically and sustainably important agronomic parameters - cropping intensity and crop diversity - have been marginal, at best, if not negative (though statistically insignificant) (Datta, 2015).

**Table 7:** ATT for outcome variables of interest.

Variable	outcome	Treated	Controls	Difference	S.E	T-stat
Crop income	Unmatched	15978.76	10303.46	5675.299	879.81	6.45∗∗∗
	ATT	13108.86	12687.02	421.85	1004.41	0.42

##### 3.2.4.1 Sensitivity analysis of ATT estimation

The results of the sensitivity analysis are presented in Table 8. According to the Rosenbaum limits sensitivity test, soil and water conservation has a positive and statistically significant impact on the income of households producing teff and sorghum crops (at **the 1** % significance level) (Rosenbaum, 2002). This indicated that unobserved confounders had no impact on the results. The robustness checks contrast the estimates from several kernel matching estimators and regressions, showing that the final crop income estimate is consistent with the primary matching results, implying that our estimate is reliable.

**Table 8:** Sensitivity analysis using Rosenbaum bounding approach.

Gamma	sig+	sig-	t-hat+	t-hat-	CI+	CI-
1	0	0	12500	12500	11575	13475
1.25	0	0	11775	13200	10875	14200
1.5	0	0	11250	13825	10275	14800
1.75	0	0	10750	14325	9800	15325
2	0	0	10325	14750	9400	15775
2.25	0	0	10000	15100	9012.5	16250
2.5	0	0	9675	15450	8700	16650
2.75	2.20E-16	0	9425	15750	8450	17000
3	3.00E-15	0	9175	16025	8200	17325

## 4 Conclusion

Recently, the role of soil and water conservation initiatives in increasing agricultural income and improving the lives of smallholder farmers has received increasing attention. However, empirical evidence on the actual impact of watershed interventions on farmers’ income and livelihoods is lacking. This study examined how soil and water conservation methods affect crop income (teff and sorghum). The estimated results showed that participants in soil and water conservation programs had higher crop yields than their counterparts did. Estimates of the propensity score matching model using the kernel matching algorithm showed that soil and water conservation programs have a beneficial impact on agricultural income. Extension contact, training, distance from the farmer’s home to a farmer’s training center, total acreage owned, livestock ownership, and household age were found to be the most important factors influencing farmer engagement in soil and water conservation practices. Participation in the program resulted in a 422 ETB increase in the total household income. Based on the findings of this study, we propose that governmental and non-governmental development partners invest in farmer capacity building through extension and training to improve soil and water conservation goals, while also addressing the livelihood concerns of resource-dependent local farmers.

## Declarations

### Author contribution statement

Seyfu Tesfayohannes & Getahun Kassa: Conceived and designed the experiments; Performed the experiments; Analyzed and interpreted the data; Contributed reagents, materials, analysis tools or data; Wrote the paper.

Yared Mulat: Conceived and designed the experiments; Analyzed and interpreted the data; Wrote the paper.

### Funding statement

This research did not receive any specific grant from funding agencies in the public, commercial, or not-for-profit sectors.

### Data availability statement

Data will be made available on request.

### Declaration of interest’s statement

The authors declare no conflict of interest.

### Additional information

No additional information is available for this paper.

## APPENDIX

### Propensity Score Matching for Impact Evaluation

The PSM method evaluates the treatment effect for all situations where one group is the treatment individual and another group is the untreated individual. It is useful when the treatment (participation in soil conservation program) is not by random assignment but depends stochastically on the observed characteristics. The steps in PSM include: the estimation of the propensity score with the Logit model, the second step checking for common support regions, the third step is the selection of the best matching algorithm, the fourth step balancing test, the fifth step is an estimation of the average treatment effect (ATT), and the final step is conducting sensitivity analysis.

### Model Specification for PSM

A Logit model was constructed over probabilities to estimate propensity scores using a pre-intervention characteristic of the sampled households. In estimating the Logit model, the dependent variable was SWC program participation status, which takes the value of 1 if a household participated and 0 otherwise. Accordingly, the probability of being in the SWC program (Pi) is:-

(1) $$Pi=\frac{e^{zi}}{1+e^{zi}}$$

The probability that a household belongs to a non-program (1-Pi) is

(2) $$1-Pi=\frac{1}{1+e^{zi}}$$

The odds ratio $\left(\frac{pi}{1-pi}\right)$ is defined as:

(3) $$\frac{pi}{1-pi}=\frac{1+e^{zi}}{1+e{-}^{zi}}=e^{zi}$$

And, L, the Logit model was given by the following relationships;

(4) $$Li=\ln\left(\frac{pi}{1-pi}\right)=Zi=\beta 1+\beta 2Xi+Ui$$

Where, *P*_*i*_ is the probability of participation, e = 2.71828, *i* = 1, 2, 3, ----, *n*.

*β*_*1*_ = intercept,
*β*_*2*_ = regression coefficients to be estimated,
*X*_*i*_ = pre-SWC program intervention characteristics and
U_i_ = an error term.

Therefore, the standard framework in evaluation analysis to formalize this problem, that is the potential outcome approach or Roy–the Rubin model (Roy, 1951; Rubin, 1974) has been used. The potential outcomes were then defined as Yi (Di) for each individual i, where Yi = outcome variable, i = 1… N and N denotes the total population, whereas, the treatment effect (TEi) for individual i was calculated using

(5) TEi=Yi(1)−Y(0)

From Eq. (5), two parameters (ATE and ATT) are the most frequently estimated.

The first one is the population average treatment effect (ATE), which is the difference between the expected outcomes after participation and non-participation in the given program given by:

(6) τATE=E(τ)=E[Y(1)−Y(0)]

Second, as stated by Rosenbaum and Rubin (1983), if the exposure to treatment is random within cells defined by X, it is also random within cells defined by the values of the mono-dimensional variable p(X) given by the following relationships.

$$P\left(X\right)=\text{Pr}\left\{D=\;\frac{1}{X}\right\}=E\left\{\;\frac{D}{X}\right\}$$

p(X) = Pr{D = 1 |X} = E{D |X}, where D = {0,1}is the indicator of exposure to treatment (variable for treatment group selection) and X is the multidimensional vector of pre-treatment characteristics. Propensity scores, therefore, describe the likelihood that a population member would have been selected for the treatment group based on a set of model variables, given that they were eligible. As a result, given a population of units denoted by i, if the propensity score P(Xi) is known, the ATT was estimated as follows:

(7) $$ATT=E\left\{Y1i-\left(\;\frac{Y0i}{Di}\right)=1\right\}$$

$$\;=E\{E\left\{Y1i-\left(\;\frac{Y0i}{Di}\right)=1,P\left(Xi\right)\right\}\}$$

$$\;=E\left\{E\left\{\left(\;\frac{Y0i}{Di}\right)=1,P\left(Xi\right)\right\}-E\{\;\frac{Y0i}{Di}=0,\;\frac{P\left(Xi\right)}{Di}\right\}=1$$

Where, the outer expectation is over the distribution of $\;\frac{P\left(Xi\right)}{Di}=1$ and Y1i and Y0i are the potential outcomes (crop income) in SWC program participation and non-participation groups respectively.

### Checking common support region

The common support region for the estimated propensity score is constructed based on comparing the minima and maxima of the propensity score in the treated and non-treated groups. The basic criteria of this approach are to delete all observations whose propensity score is smaller than the minima and larger than the maximum in the opposite group (Caliendo and Kopeinig, 2008).

### Choice of matching algorithm

The most widely used methods to match observations based on the closeness of PSM are:-

**Nearest neighbor matching (NNM)**:- ensures that each treated observation is considered by matching it with a control observation having the closest propensity score. For this algorithm, the bandwidth used were 1, 2, 3, 4, and 5.

**Calipers or Radius:-**In this method each treated observation is matched with those control observations that fall within a pre-specified neighborhood (radius) of the PS of the treated observation. Here, the size of the radius plays an important role. If it is set to be very small, some treated observations may not be considered because they may not find a match from the control. However, lesser radius sizes might result in better matches. For this study, radius matching was tested with caliper sizes of 0.01, 0.1, 0.25, and 0.5.

**Kernel matching: -** All treated observations are matched with a weighted average of all control observations with weights that are inversely proportional to the distance between the propensity scores of treated and control groups. It’s tested with bandwidths of 0.01, 0.1, 0.25, and 0.5.

### Testing matching quality

Choice of matching algorism was made based on the criteria such as a large number of the insignificant variables after marching, a large number of the matched sample size, and low pseudo R^2^ after matching which balances all explanatory variables (i.e., results in insignificant mean differences between the two groups), low standard mean bias (3.5%–5%) (Caliendo and Kopeinig, 2008).

**Standardized bias (SB):** This is one suitable indicator used to assess the distance in marginal distributions of the *X* variables. It helps to quantify the bias between control and treated groups. It was done before and after matching. The formula by Caliendo and Kopeinig (2008) is.

Standardized bias (SB) before matching:-

(8) $$\text{SBbefore}=\frac{\text{xt}-\text{xc}}{\sqrt{0.5\left(\text{vt}\left(\text{x}\right)+\text{vc}\left(\text{x}\right)\right)}}\text{∗}100$$

Where, Xt (Vt) and Xc (Vc) represents a mean (variance) in the treatment and control groups before matching, respectively.

### Standardized bias (SB) after matching:-

(9) $$\text{SBafter}=\frac{\text{xtm}-\text{xcm}}{\sqrt{0.5\left(\text{vtm}\left(\text{x}\right)+\text{vcm}\left(\text{x}\right)\right)}}\text{∗}100$$

Where Xtm (Vtm) and Xcm (Vtm) represent a corresponding value for matched samples. One problem with the standardized bias approach is that there is no clear sign of the success of the matching procedure. However, Rosenbaum and Rubin (1983) argue that after matching, a total bias of over 20 percent is large. The bias reduction (BR) is computed as:

(10) $$BR=\left(1-\frac{B\left(x\right)\;after}{\;B\left(x\right)before}\right)*100$$

Where, B (x) _after_, B (x) _before_ are total bias after and before, respectively.

**T-test:** As described by Rosenbaum and Rubin (1983), a two-sample t-test was applied to check if there is a significant difference between the variables’ means of both groups. Before matching, the difference is expected, but after matching, the variables are balanced and there should be no significant difference between the two groups.

**Joint significant and pseudo R**^**2**^: the pseudo-R^2^ demonstrates how well the regression X’s explain the probability of participation. It’s expected that after matching there should be no statistical difference in the distribution of treatment and control group and hence the pseudo-R^2^ after matching is fairly low (Caliendo and Kopeinig, 2008).

### Sensitivity analysis

The final step in the implementation of PSM is checking the sensitivity of the estimated (Caliendo and Kopeinig, 2008). The matching method is based on the CIA, which states that the evaluator should observe all variables that are simultaneously influencing the participation decision and outcome variables. Since matching estimators are not robust against hidden biases, it’s important to test the robustness of the result to departure from the identifying assumptions. However, it’s impossible to estimate the magnitude of selection bias with non-experimental data. Therefore, this problem can be addressed by sensitivity analysis (Caliendo and Kopeinig, 2008). To check the sensitivity of the estimated ATE concerning deviation from the CIA, it’s suggested that the Rosenbaum bounding approach is appropriate (Rosenbaum and Rubin, 1983).

## Appendix I Supplementary data

Questionnaire SWC.docx Questionnaire SWC.docx
