# Supplementary material for: Impact of soil and water conservation practices on crop income in tembaro district, southern Ethiopia
Source: Heliyon. 2022 Aug 10;8(8):e10126. doi: 10.1016/j.heliyon.2022.e10126 (PMC9396550; doi:10.1016/j.heliyon.2022.e10126)
Supplement: Questionnaire SWC.docx [file mmc1.docx]

**Household survey questionnaire SWC**

**Research Title:-** Impact of SWC on Crop Income in Tembaro District, Southern Ethiopia.

Zone …………..……*District* ……..……*kebele*……………………..*village*…………..……

Name of the enumerator…………..………..............… Signature…..…… date…………..……

1. General socio-economic information
   1. Name of household head……………………. Age…………
   2. Sex: 1. Male 0. Female
   3. Marital status: 1. Married 2. Single 3.Divorce 4. Widowed
   4. Total family size….……1. Male…………. 2. Female ………………
   5. Category of family size 1. Age< 15yrs…..…2. Age15-64yrs...……3. Age above 64…
   6. Education level in grade …………what is your status now?1) can read and Wright 2) cannot read and right
   7. Farming experience………………years
   8. Distance from home to the farm land .............in (km)................in (hr)?
   9. Distance from home to FTC …………..in (km)………in (hr)?
2. **Land holding Owing in 2019/2020 G.C.**
   1. Total land holdings in (timad)……Cultivated……Grazing……Fallow ……other…..
   2. No of plots owned………
   3. Slope of the land 1. Very steep 2. Steep. 3. Gentle slope. 4. Flat
3. **Perception on the SWC practice in the area and its impacts.**
   1. Did you participate in SWC practice provided by SARI? 1) yes 2) no
   2. If yes, which type 1. Physical SWC practice 2. Biological SWC practice 3. Both
   3. In which kind of soil conservation programs have you been involved? 1. Food for work 2. Money for work 3. Free 4. Others (Specify)
4. **Livestock Ownership 2019/2020 G.C**
   1. Do you have livestock? 1. Yes 0. No, if yes, number owned? Cows……..…Oxen……..… Heifer……..… Calves……..… Sheep……..Goat……..… Donkey……..…Horse……..… Mule……..… Poultry……..…
5. **Cropping pattern (2019/20G.C)/ 2012E.C**

| Major Crops grown | Area covered in ha | Yield obtained in qt | Amount consumed in qt | Amount sold in qt | Income earned from selling in Birr |
| --- | --- | --- | --- | --- | --- |
|  |  |  |  |  |  |
|  |  |  |  |  |  |
|  |  |  |  |  |  |

- 1. Have you noticed an increase in production from these major crops? 1. Yes 2. No
  2. If is yes, what do you think is /are the main reasons for the increase in crop production? 1. Better effort (labor productivity) 2. Improved cultivation practice 4. Improved soil and water conservation practice 5. Other specify.
  3. If no, what do you think is/are the main problem if not increased? 1. Soil erosion problem 2. Labor productivity problem 3. Technology usage problem (row planting, new varieties) 4. Other

1. **Amount and cost of fertilizer and chemicals used for the crop.**

| Type of crop to which fertilizer applied | NPS (kg) | Urea (kg) | Cost of fertilizer (Birr) | Manure in (kg) | Compos in (kg) | Cost of chemicals applied (Birr) |
| --- | --- | --- | --- | --- | --- | --- |
| Teff |  |  |  |  |  |  |
| Others |  |  |  |  |  |  |

1. **Animal forage related questions**
   1. Did you grow any animal forage in your farm? 1. Yes 2. No if yes,
   2. Which type? 1. Elephant grass 2. Desho grass 3. *sesbania* and lucinia 4. *Acacia decurrens* and *acacia* saligina 5. All
   3. What is the purpose of growing? 1. for feeding livestock 2. For soil water conservation 3. For sale as a source of income 4. 1 and 2 5. All
2. **Income generated from farming and non/off-farm activities**
   1. Did you have any other source of income other than agriculture? 1. Yes 2. No
   2. What are your main sources of income? 1. Farming 2. Off-farm 3.both
   3. Income from (crop……Livestock sell…… Dairy products……Fruit and related……… animal forage selling….. Remittance… Non-farm (daily labor (other than PSNP), petty trade, handcraft, etc,)…… PSNP if user……) in Birr. Total income……..Birr.
3. **Social Responsibility**
   1. Do you have any responsibility in the community? 1. Yes 0. No
   2. If yes, what is your responsibility in the community? 1. Administrative 2. Social 3.Religious 4. Watershed committee member All
   3. Do your responsibilities have a negative impact in farming activities? 1. Yes 0. No
4. **Credit access**
   1. Is there a credit access in your area? 1. Yes 2.no
   2. Have you got credit in the past three years 2019/20? 1. Yes 0. No
   3. If yes, how much have you borrowed in the last two years ……birr From microfinance…….. Cooperatives ………. Relatives…… local money lenders……
   4. Did you started to repay 1) if yes 2) no if yes, how much did you repaid back……...
   5. If it is not totally repaid, why? ......................................................................................
5. **Extension Service**
   1. Number of contacts with development agents per year………………………
   2. Have you been advised by any development agent about SWC practices? 1. Yes, 0.no
6. **Training and related service provision**
   1. . Have you got training related with soil conservation practice? 1. Yes 2. No if yes,
   2. . Who provided the training? 1. Research center 2. NGO 3. Agricultural Offices 4. Agricultural Offices and NGO
   3. . Have you been advised by any of these organizations to undertake soil conservation practices? 1. Yes, 0.no
7. **Awareness related with soil erosion**
   1. Do you perceive soil erosion problem in your area? 1. Yes 0. No
   2. If yes, what is the indication that such problem exists?

1. Productivity decrease 2. Soil depth decrease 3. Soil color changes 4.Others…………..

- 1. Is your farmland prone to erosion? 1. Yes 0. No
  2. If yes, how much of your farmland affected by erosion in --------------- (timad)
  3. How do you perceive the level of parcel’s (land) exposure to soil erosion? 1. No risk 2. Medium 3. High exposure to erosion
  4. How does the household perceive the soil erosion since starting farming as Compared to the past? 1. Increasing 2. Decreasing 3. No change 4. Do not know
  5. If increasing, what measures did you taken to rehabilitate the conditions? 1. Apply physical soil conservation method 2. Apply biological soil conservation method 3. Apply both soil conservation methods.
  6. Did you practice any of the following physical soil conservation practice(s)? 1. Terrace 2. Counter bunds 3. Grass strip 4. Soil and stone bund 5. All
  7. If you did not practice any soil conservation measures, what is the reason? 1. Lack of money 2. Labor shortage 3. Awareness problem 4.Others ………………….

1. **Questions related Labor availability**
   1. Do you have labor shortage for farm activities? Yes =1 no =0
   2. If yes, for which kind of farm activities? 1. Crop production2. Livestock production 3. Soil conservation activities
   3. If yes for the question14.1, how do you solve the problem? 1. Hiring labor 2. Use debo system 3.Other (specify)
   4. If you hire labor, how much do you pay per year? _____ Birr
